# Outcomes of laparoscopic versus open total gastrectomy with D2 lymphadenectomy for gastric cancer: a systematic review and meta-analysis

**DOI:** 10.1186/s40001-022-00748-2

**Published:** 2022-07-18

**Authors:** Yongpu Yang, Yuyan Chen, Yilin Hu, Ying Feng, Qinsheng Mao, Wanjiang Xue

**Affiliations:** 1 Department of Gastro intestinal Surgery, Affiliated Hospital of Nantong University, Nantong, 226001 Jiangsu China; 2 Research Center of Clinical Medicine, Affiliated Hospital of Nantong University, Nantong, 226001 Jiangsu China; 3 Department of Graduate School, Dalian Medical University, Dalian, 116000 Liaoning China

**Keywords:** Laparoscopic total gastrectomy, D2 lymphadenectomy, Gastric cancer, Surgical outcomes, Survival outcomes, Meta-analysis

## Abstract

**Background:**

The effectiveness of laparoscopic total gastrectomy with D2 lymphadenectomy (LTGD2) remains controversial. This meta-analysis compares surgical and survival outcomes of LTGD2 and open total gastrectomy with D2 lymphadenectomy (OTGD2) for gastric cancer.

**Methods:**

Controlled studies comparing LTGD2 and OTGD2 published before November 2021 were retrieved via database searches. We compared intraoperative outcomes, pathological data, postoperative outcomes, 5-year disease-free survival (DFS), and overall survival (OS).

**Results:**

17 studies were included, containing 4742 patients. Compared with OTGD2, the LTGD2 group had less blood loss (mean difference [MD] = − 122.48; 95% CI: − 187.60, − 57.37; P = 0.0002), fewer analgesic medication (MD = -2.48; 95% CI: − 2.69, − 2.27; P < 0.00001), earlier first flatus (MD = − 1.03; 95% CI: − 1.80, − 0.26; P = 0.009), earlier initial food intake (MD = − 0.89; 95% CI: − 1.09, − 0.68; P < 0.00001) and shorter hospital stay (MD = − 3.24; 95% CI: − 3.75, − 2.73; P < 0.00001). The LTGD2 group had lower postoperative total complication ratio (OR = 0.76; 95% CI: 0.62, 0.92; P = 0.006), incision (OR = 0.50; 95% CI:0.31, 0.79; P = 0.003) and pulmonary (OR = 0.57; 95% CI: 0.34, 0.96; P = 0.03) complication rates, but similar rates of other complications and mortality. Total number of dissected lymph nodes were similar, but the number of No. 10 dissected nodes was less with LTGD2 (MD = − 0.31; 95% CI: − 0.46, − 0.16; P < 0.0001). There was no difference in 5-year OS (P = 0.19) and DFS (P = 0.34) between LTGD2 and OTGD2 groups.

**Conclusions:**

LTGD2 produces small trauma, fast postoperative recovery and small length of hospital stays than OTGD2, and had similar long-term clinical efficacy as OTGD2. However, these results still need further high-quality prospective randomized controlled trials confirmation.

**Supplementary Information:**

The online version contains supplementary material available at 10.1186/s40001-022-00748-2.

## Introduction

Gastric cancer (GC) is one of the most common gastrointestinal tumors, and it is especially frequent in East Asia. New cases of GC in China account for 2/5 of all new cases across the world annually [1]. Radical surgery remains an important method of treatment for GC, but overall prognosis is relatively poor. In recent years, morbidity due to GC has been increasing. According to Japanese treatment guidelines for gastric cancer established by the Japanese Gastric Cancer Association, patients with upper- and middle-third GC, as well as large gastric tumors, should be treated with total gastrectomy (TG), accompanied by an appropriate extent of lymphadenectomy. Traditional open total gastrectomy (OTG) is the most popular and extensively used form of TG. However, OTG cannot meet patients’ increasing requests for painless, non-invasive surgery because it involves a long surgical incision, extensive surgical trauma due to strong intraoperative traction, substantial postoperative pain, and slow postoperative recovery [2].

In 1994, Kitano et al. initially reported the use of laparoscopic gastrectomy in the treatment of early GC in Japan [3]. With the increasing publication of high-quality evidence, laparoscopy has been used more extensively in the treatment of early GC and has demonstrated favorable short- and long-term clinical efficacy, similar to that achieved with open gastrectomy. In the 4th edition of Japanese Gastric Cancer Treatment Guidelines, laparoscopic distal gastrectomy (LDG) has been considered as one of accepted treatments for patients with Stage I GC. In 1999, Azagra et al. reported the surgical safety and feasibility of laparoscopic total gastrectomy (LTG), accompanied by differing extents of lymphadenectomy [4], in 13 patients with GC.

Although laparoscopic radical surgery is an accepted treatment for early GC, it still remains controversial in treating advanced GC, especially LTG combined with D2 lymphadenectomy (LTGD2) [5, 6]. Multiple single-center retrospective studies have demonstrated that LDG combined with D2 lymphadenectomy (LDGD2) is a feasible and safe non-invasive technique. Compared with OTGD2, LTGD2 has higher operative requirements and involves a larger extent of surgery, more complex gastrointestinal reconstruction, and higher risk of seeding and metastasis of detached cancerous cells [7]. However, few reports have been published regarding long-term survival outcomes of LTGD2.

This study aims to compare surgical and survival outcomes of LTGD2 versus OTGD2 for the treatment of GC through meta-analysis of the available literature to provide objective and reliable evidence-based rationale for the use of LTGD2 in GC.

## Material and methods

### Ethics statement

This study was deemed exempt from institutional review board approval by Nantong University Affiliated Hospital, and informed consent was waived. We conducted this study in accordance with the ethical standards of the World Medical Association Declaration of Helsinki.

### Literature retrieval

Studies published in English before November 2021 were retrieved from electronic searches of PubMed, Elsevier, Cochrane Library, and OVID databases. We used these key words: GC, stomach neoplasms, gastric tumor, laparoscopic, laparoscopy, TG, total gastric resection, D2 dissection, and D2 lymphadenectomy.

### Inclusion and exclusion criteria

The study inclusion criteria were as follows: any type of controlled study; LTGD2 was compared to OTGD2 for the treatment of GC; LTGD2 included hand-assisted, laparoscope-assisted, or total laparoscopic surgery methods; data were presented for surgical safety and efficacy, as well as means and standard deviations of the constant variables; and the study had a quality score of > 5 points according to the Newcastle–Ottawa scoring system (NOS) [8].

The study exclusion criteria were as follows: enrollment of patients without GC; non-D2 lymphadenectomy or partial gastrectomy were included in the study, unless the data were presented separately for TG with D2; no data directly compared LTGD2 and OTGD2; received neo-adjuvant chemotherapy (NAC) and adjuvant chemotherapy; conversion to laparotomy; robot-assisted surgery; and reviews or meta-analyses.

### Data collection

Risk of bias of the included studies was assessed using the risk of bias algorithm outlined by the Cochrane Collaboration Handbook, and data were collected by two authors independently [9]. A third author was invited to resolve disputes, when necessary. The collected clinical data included surgical duration, intraoperative blood loss, analgesic use, time of first flatus, time of initial food intake, length of hospital stay, proximal resection margin, distal resection margin, total number of dissected lymph nodes, number of No. 10 dissected lymph nodes, mortality within 30 days postoperatively, occurrence of postoperative complications (including stomal leak, anastomotic stenosis, gastrointestinal hemorrhage, abdominal hemorrhage, pulmonary complications, incision complications, abdominal infection, intestinal obstruction, duodenal stump fistula, and pancreatic fistula), and long-term clinical efficacy (5-year disease-free survival [DFS] and overall survival [OS]).

### Statistical data analysis

RevMan 5.4 software was used for the meta-analyses. The summary statistic used for second-classification data was the odds ratio (OR), and the summary statistic used for constant variable data was the weighted mean difference (MD). The results are expressed by 95% confidence interval (CI), and a forest plot was used to evaluate statistical significance. Heterogeneity of the included studies was assessed using the *I*^*2*^ test. Heterogeneity was considered significant if *I*^*2*^ was 50% or greater. Random effect models were used when heterogeneity was significant; otherwise, fixed effect models were used. Funnel plots were used to evaluate possible publication bias.

## Results

### Number and quality of included studies and publication bias

**Fig. 1:**
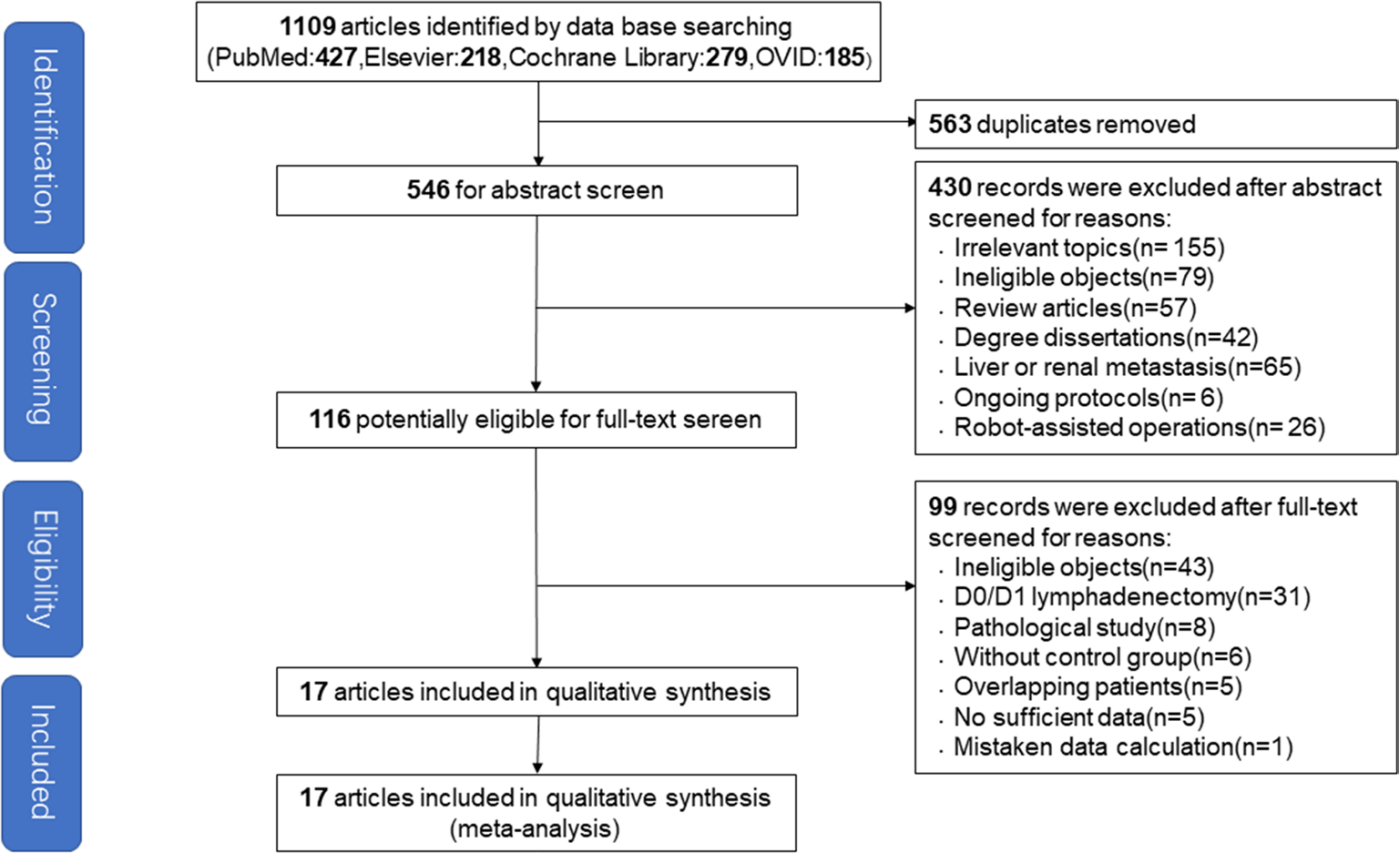
Flowchart describing the study selection process

A total of 1,109 studies comparing LTGD2 and OTGD2 were initially retrieved; 546 remained after excluding duplicate studies. After screening the abstracts of these 546 articles, the full texts of 116 potentially relevant studies were reviewed. Of these, 17 studies were included in the current study based on inclusion and exclusion criteria (Fig. 1) [6, 10–17].

**Fig. 2:**
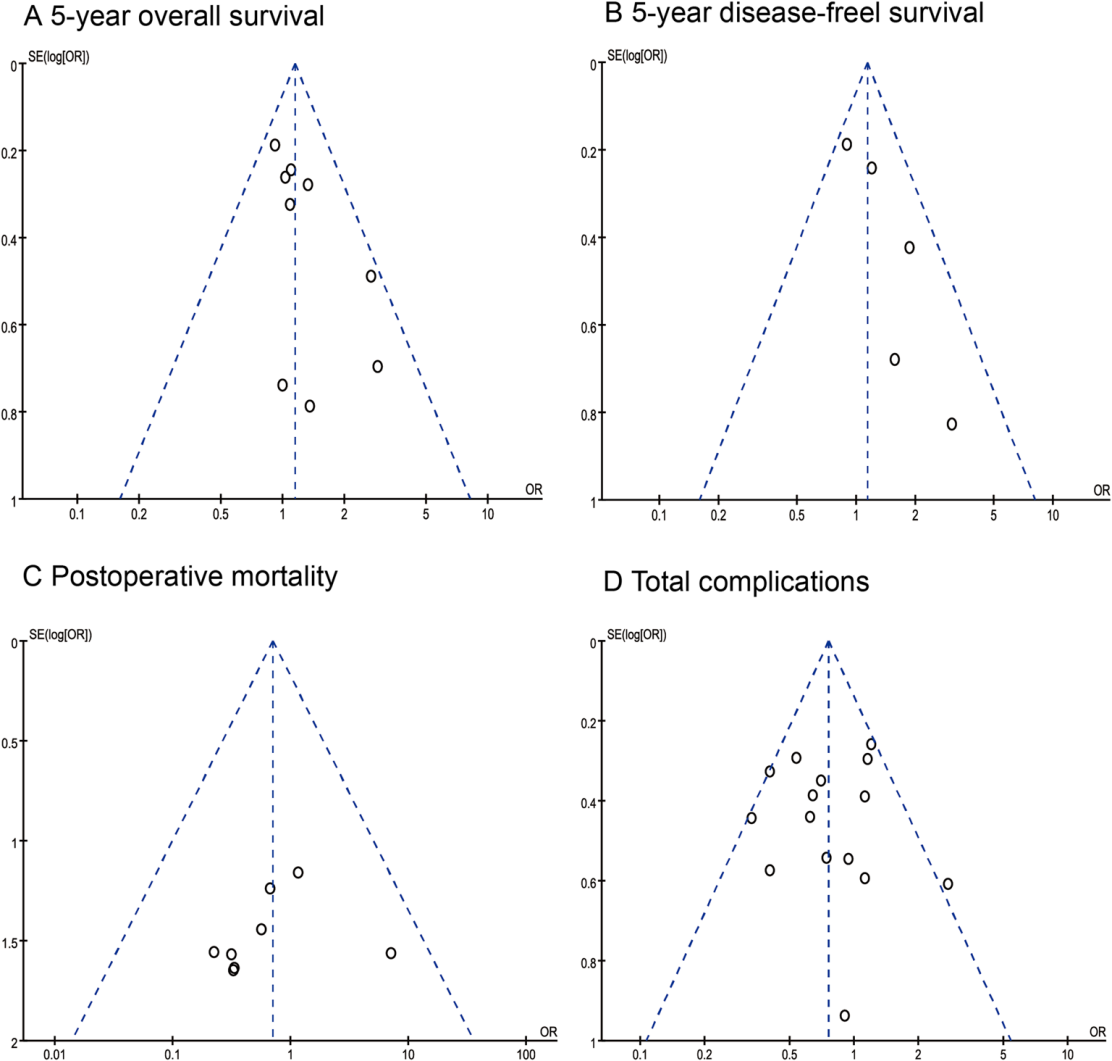
Funnel plots of each outcome. (A) 5-year overall survival, (B) 5-year disease-free survival, (C) postoperative mortality, and (D) total complications

The quality assessment results (NOS score) for each study are summarized in Additional file 1: Table S1. According to the funnel plots, there was no evidence of significant publication bias in this meta-analysis (Fig. 2). When significant heterogeneity was found, we eliminated the articles responsible for the heterogeneity and re-performed the meta-analysis.

### Characteristics of included studies

From the 17 included studies, which were published from 2008 to 2021 (Additional file 1: Table S1), a total of 4742 patients were enrolled in this meta-analysis: 1993 underwent LTGD2 and 2749 underwent OTGD2 (Additional file 1: Table S1). Most studies included both early and advanced GC. When considering all studies, there were no significant differences in patient age, body mass index (BMI), size and site of tumor, American Society of Anesthesiologists’ class, or distribution of tumor node metastasis (TNM) stages between the LTGD2 and OTGD2 groups (P > 0.05; Additional file 1: Table S1).

### Intraoperative and postoperative recovery

Compared with the OTGD2 group, the LTGD2 group had less blood loss (MD = − 122.48; 95% CI: − 187.60, − 57.37; P = 0.0002), fewer days of analgesic medication (MD = − 2.48; 95% CI: -2.69, − 2.27; P < 0.00001), earlier time of first postoperative flatus per rectum (MD = − 1.03; 95% CI: − 1.80, − 0.26; P = 0.009), earlier time of initial food intake (MD = − 0.89; 95% CI: − 1.09, -0.68; P < 0.00001) and shorter length of postoperative hospital stay (MD = − 3.24; 95% CI: − 3.75, − 2.73; P < 0.00001; Additional file 2: Figure S1). However, the LTGD2 group had a longer operation time (MD = 40.44; 95% CI: 27.41, 53.47; P < 0.00001). Subgroup analysis was performed for operation time based on the year of study publication. These results showed that the LTGD2 group had a longer operation time than the OTGD2 group for the 5 studies published before 2013 (MD = 42.53; 95% CI: 16.23, 68.82; P = 0.002), as well as the 6 studies published after 2013 (MD = 39.29; 95% CI: 25.05, 53.49; P < 0.00001; Additional file 2: Figure S1F).

### Pathological data

**Fig. 3:**
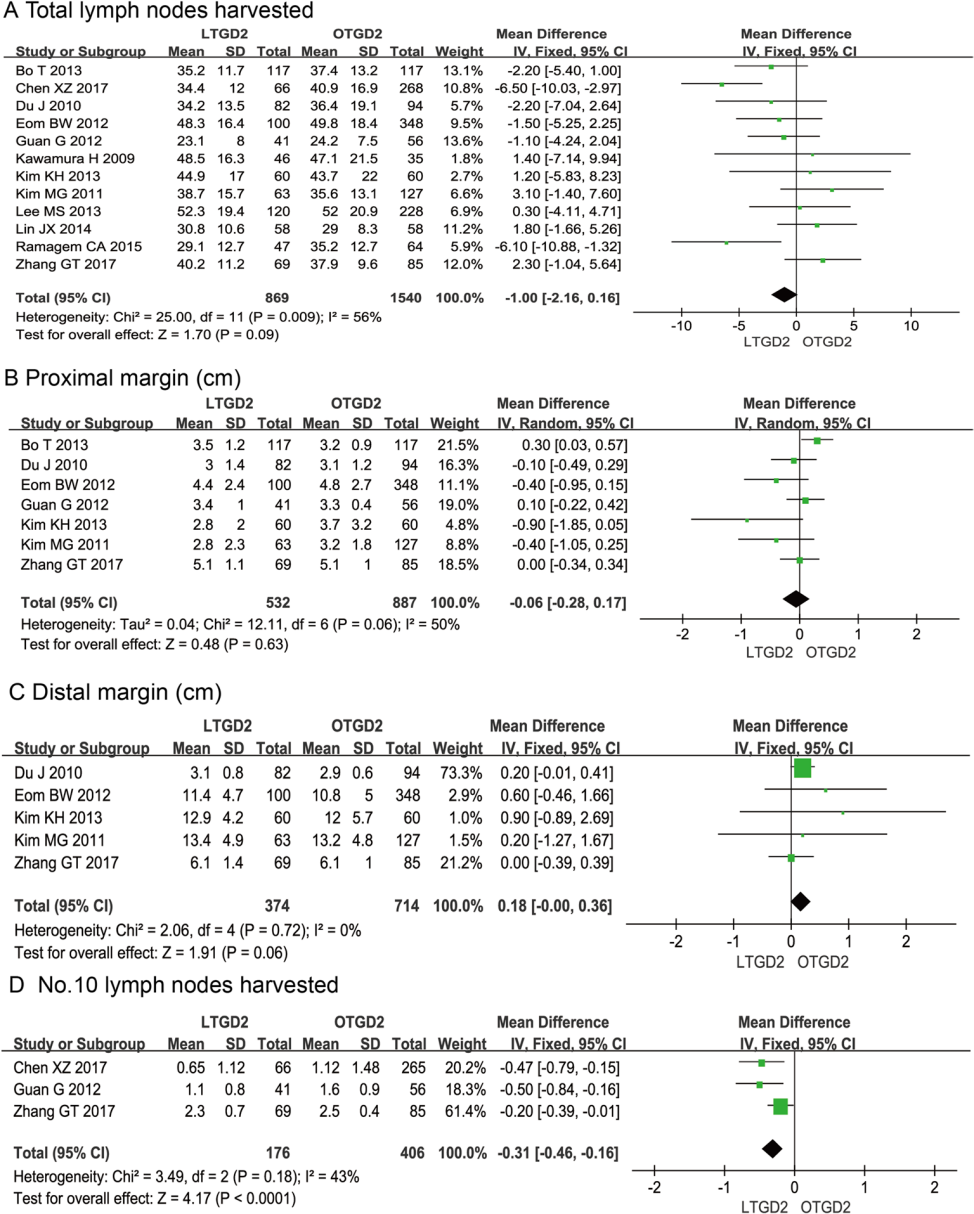
Analysis comparing (A) total number of harvested lymph nodes during D2 dissection, (B) proximal resection margin, (C) distal resection margin, and (D) number of No. 10 harvested lymph nodes. LTGD2, laparoscopic total gastrectomy with D2 lymphadenectomy; OTGD2, open total gastrectomy with D2 lymphadenectomy

There were no significant differences between LTGD2 and OTGD2 groups with regard to the total number of dissected lymph nodes (MD = − 1.00; 95% CI: − 2.16, -0.16; P = 0.09; Fig. 3A), proximal resection margin (MD = − 0.06; 95% CI: − 0.28, 0.17; P = 0.63; Fig. 3B), and distal resection margin (MD = 0.18; 95% CI: − 0.00, 0.36; P = 0.06; Fig. 3C). However, the number of No. 10 dissected lymph nodes was significantly less in the LTGD2 group than in the OTGD2 group (MD = − 0.31; 95% CI: − 0.46, − 0.16; P < 0.0001; Fig. 3D).

### Postoperative mortality and complications

The postoperative mortality rate was similar in the LTGD2 and OTGD2 groups (OR = 0.70; 95% CI: 0.29, 1.73; P = 0.44; Additional file 2: Figure S2A). The rate of total postoperative complications was less in the LTGD2 group than in the OTGD2 group (OR = 0.76; 95% CI: 0.62, 0.92; P = 0.006; Additional file 2: Figure S2B). Subgroup analysis of specific types of postoperative complications found no significant differences between groups for anastomotic leakage (OR = 0.94; 95% CI: 0.61, 1.47; P = 0.80), anastomotic stenosis (OR = 2.01; 95% CI: 0.81, 4.99; P = 0.13), anastomotic bleeding (OR = 0.52; 95% CI: 0.20, 1.36, P = 0.18), abdominal hemorrhage (OR = 0.87; 95% CI: 0.33, 2.27; P = 0.29), abdominal infection (OR = 0.59; 95% CI: 0.28, 1.23; P = 1.41), intestinal obstruction (OR = 1.54; 95% CI: 0.81, 2.92; P = 0.19), internal hernia (OR = 1.53; 95% CI: 0.21, 11.08; P = 0.68), lymph leakage (OR = 0.46; 95% CI: 0.10, 2.13; P = 0.32), and pancreatic fistula (OR = 0.74; 95% CI: 0.22, 2.56; P = 0.64; Table 1). However, LTGD2 group had notably lower rates of postoperative complications related to the incision (OR = 0.50; 95% CI: 0.31, 0.79; P = 0.003; Additional file 2: Figure S2C) and pulmonary complications (OR = 0.57; 95% CI: 0.34, 0.96; P = 0.03; Additional file 2: Figure S2D) than the OTGD2 group.

**Table 1:** Postoperative complications comparing LTGD2 with OTGD2

Items	OR (95%CI)	Test for overall effect		Test for heterogeneity	
		Z	P	I^2^(%)	P
Anastomotic leakage	0.94(0.61,1.47)	0.25	0.80	0	0.66
Anastomotic stenosis	2.01(0.81,4.99)	1.51	0.13	0	0.84
Anastomotic bleeding	0.52(0.20,1.36)	1.34	0.18	0	0.80
Abdominal hemorrhage	0.87(0.33,2.27)	0.29	0.78	0	0.64
Lymphorrhagia	0.46(0.10,2.13)	1.00	0.32	51	0.15
Abdominal infection	0.59(0.28,1.23)	1.41	0.16	24	0.26
Intestinal obstruction	1.54(0.81,2.92)	1.32	0.19	0	0.70
Internal hernia	1.53(0.21,11.08)	0.42	0.68	0	0.34
Pancreatic fistula	0.74(0.22,2.56)	0.47	0.64	0	0.80

*CI* confidence interval, *LTGD2* laparoscopic total gastrectomy with D2 lymphadenectomy, *OR* odds ratio, *OTGD2* open total gastrectomy with D2 lymphadenectomy

### Long-term clinical efficacy

**Fig. 4:**
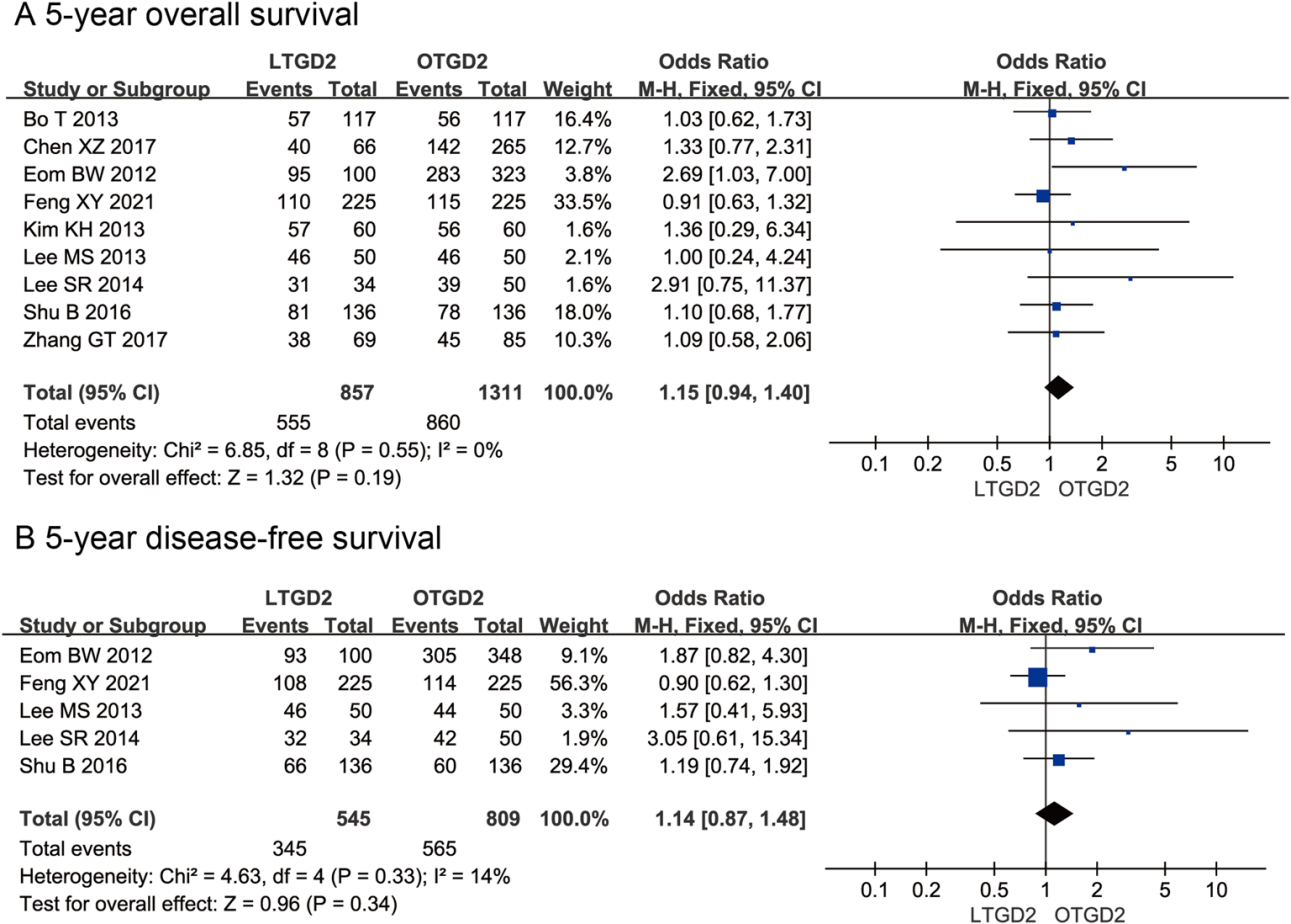
Analysis comparing (A) 5-year overall survival, including subgroup analysis according to dissection of No. 10 nodes; and (B) 5-year disease-free survival. LTGD2, laparoscopic total gastrectomy with D2 lymphadenectomy; OTGD2, open total gastrectomy with D2 lymphadenectomy

Survival data were available for seven included studies. The pooled analysis showed there is no significant difference in 5-year OS (OR = 1.15; 95% CI: 0.94, 1.40; P = 0.19; Fig. 4A) and DFS (OR = 1.14; 95% CI: 0.87, 1.48; P = 0.34; Fig. 4B) between the LTGD2 and OTGD2 groups.

## Discussion

This meta-analysis found that LTGD2 was associated with a markedly longer surgical duration than OTGD2, but there were no significant differences between groups in the rates of total postoperative complications and postoperative mortality. Furthermore, the LTGD2 group had less intraoperative blood loss and fewer postoperative analgesic injections, time of initial postoperative flatus per rectum, time of initial food intake, and shorter length of postoperative hospital stay, when compared with the OTGD2 group. These results indicate that LTGD2 has substantial advantages over OTGD2 with regard to postoperative recovery of patients with GC. In addition, we found that there were no significant differences between two LTGD2 and OTGD2 in the total number of dissected lymph nodes, proximal resection margin, and distal resection margin. Although the number of dissected No. 10 lymph nodes was fewer in the LTGD2 group, there were no significant differences between groups for 5-year OS and DFS, indicating that LTGD2 and OTGD2 had similar long-term clinical efficacy.

Of the 16 enrolled studies, 14 were from East Asia (1 from Japan, 6 from South Korean, and 7 from China), 1 was from Brazil in South America, 1 was from Europe, and none were from North America. This is consistent with the distribution of GC. D2 lymphadenectomy has been established as the current standard for treating patients with advanced GC in a number of countries, including China, Japan, and South Korea [18, 19], but no published prospective randomized controlled trials (RCTs) have demonstrated better survival with D2 lymphadenectomy than D1 lymphadenectomy in Western countries [20, 21]. Furthermore, some western study results suggest that survival is similar with D1+ and D2 lymphadenectomy, whereas surgical risks and difficulty are much less with D1 + lymphadenectomy [22, 23]. In addition, experts generally agree that an increased BMI increases the risks of laparoscopic GC surgery with D2 lymphadenectomy [24, 25]. D1+ lymphadenectomy is generally preferred in Europe and America for treating patients with advanced GC, and D2 lymphadenectomy is performed infrequently in these countries. In addition, studies have shown that compared with OTGD2, minimally invasive surgery for locally advanced gastric cancer will not harm the overall short-term mortality and morbidity. Moreover, studies have further shown that laparoscopic gastrectomy for advanced gastric cancer is better in terms of short-term and long-term outcomes [26–30].

Although LTGD2 has the advantages over OTGD2 of less surgical trauma and faster recovery, our results indicated that the LTGD2 group had a notably longer surgical duration. The degree of familiarity of surgeons with laparoscopic instruments and the degree of coordination with their assistants are two important factors influencing LTGD2 surgical duration [31]. A study involving experienced surgeons reported no significant differences in duration between LDGD2 and ODGD2 [25, 32]. The technical difficulty of mini-incision or total laparoscopic esophagojejunostomy is also an important cause of the long surgical duration of LTGD2. In mini-incision surgery, it is difficult to create the purse-string suture and imbed the anastomosis through the small incision, and it is difficult to complete the esophagojejunostomy because of the small incision and limited operative field. With total laparoscopy, it is also difficult to create the purse-string suture at the esophageal stump, and improper anastomosis may lead to severe or even fatal complications [33]. To overcome these problems, numerous new techniques are emerging, including use of a transorally inserted anvil device (OrVil^™^ system) to perform end-to-side esophagojejunostomy or an endoscopic linear stapler to conduct side-to-side esophagojejunostomy [34]. These two methods not only eliminate the need for a small upper abdominal incision, but also avoid the laparoscopic purse-string suture, which greatly shortens the surgical duration. With the accumulation of surgical experience and development of new instruments and techniques, differences in surgical duration between LTGD2 and OTGD2 will likely decrease. Despite the longer duration of LTGD2 at present, our results indicate that this was of little clinical importance, as it did not negatively influence postoperative recovery.

Postoperative mortality and complications are important indices for assessing surgical feasibility and postoperative short-term clinical efficacy. The current meta-analysis found that postoperative mortality was low and similar in both groups. TGD2-related complications mainly included stomal leak, anastomotic stenosis, anastomotic bleeding, duodenum stump fistula, and abdominal hemorrhage. TGD2-related complications have differed among studies because of different surgeons’ experiences and varied definitions and classification of complications [5, 16, 17, 24–32, 34–39]. In the current meta-analysis, the rate of total postoperative complications was less in the LDGD2 group than in the ODGD2 group. The rates were similar between groups for most complications, but the LTGD2 group had markedly lower rates of incision-related and pulmonary postoperative complications. The lower rates may be attributed to the shorter abdominal incision with LTGD2, which facilitates postoperative coughing and mobility, accelerates recovery, and shortens the hospital length of stay. Several previous meta-analyses have also shown that LTGD2 has improved short-term results, especially in terms of length of stay in laparoscopic surgery, which is balanced with the time-consuming and technical challenges of laparoscopic surgery  [40, 41].

Long-term survival rates are another index of surgical advantages and disadvantages. LTGD2 can only replace OTGD2 if it achieves long-term survival rates that are similar to those of OTGD2. Some studies have shown that LTGD2 exhibits not only radical efficacy similar to that observed with ODGD2, but also similar 5-year survival rates [5, 6, 11–17]. Lin et al. found no significant differences between LTGD2 and OTGD2 during median follow-up of 24.0 months [42]. Our current results also demonstrated that LTGD2 could provide comparable 5-year OS and DFS as OTGD2, further indicating that LTGD2 is safe and feasible for GC.

This meta-analysis was limited by the retrospective nature, small sample size, and short follow-up of most of the included studies. There was also heterogeneity of results between studies. This heterogeneity may be due to several factors, including differences in expertise of the surgeons, pathological stages of the tumors, clinical pathways, methods of measuring outcomes, and patient populations. With respect to the surgeons’ expertise, LTGD2 lacks a mature quality control system, which contributes to interregional gaps in the development of technical skills for this surgery. Furthermore, the learning curve varies for surgeons with different levels of experience [31, 37], and surgeons participating in the studies may be in different stages of the learning curve; thus, the skills of surgeons even in the same region may vary considerably [38]. Regarding pathological stages, enrolled patients may have a different TNM stage, and the ratio of patients with various TNM stages may vary between studies; advanced GC is more difficult to treat surgically than early GC. Perioperative management varies between hospitals, as there are no uniform clinical pathways for these operations. Methods of outcome measurement often vary because of lack of uniform standards for quantitative analysis of such outcomes as intraoperative blood loss or patient condition at hospital discharge. Likewise, the number of dissected lymph nodes is closely associated with the sampling experience of the pathologist. Regional variations in populations due to differences in living and diet habits, as well as body types, may also contribute to heterogeneous study results. Therefore, further prospective randomized studies from Western countries are necessary.

## Conclusion

The result of our meta-analysis demonstrates that LTGD2 is safe and feasible, with advantages of limited trauma, fast recovery, mild pain, and short length of hospital stay. However, it should be used with caution in patients with GC who have a high-risk of hilus lienis lymph node metastasis. Certainly, the results of this study require further verification by large, multi-center RCTs. Both LTGD2 and OTGD2 are reasonable surgical methods; prognosis depends mainly on the surgeon operating with a high degree of skill and safety, not on whether laparoscopic or open surgery is chosen. No matter which surgical approach is used, the most important issue is understanding the appropriate indications for TG.

## Supplementary Information

**Additional file 1: Table S1.** Details and characteristics of the studies included in this meta-analysis.

**Additional file 2: Figure S1.** Analysis comparing (**A**) blood loss, (**B**) times of analgesic medication injections, (**C**) time of first flatus per rectum, (**D**) time of initial diet, (**E**) postoperative hospital length of stay, and (**F**) subgroup analysis of operation time according to year of surgery. LTGD2, laparoscopic total gastrectomy with D2 lymphadenectomy; OTGD2, open total gastrectomy with D2 lymphadenectomy. **Figure S2.** Analysis comparing (**A**) postoperative mortality, (**B**) postoperative total complications, (**C**) postoperative incision-related complications, and (**D**) postoperative pulmonary complications. LTGD2, laparoscopic total gastrectomy with D2 lymphadenectomy; OTGD2, open total gastrectomy with D2 lymphadenectomy.

## Data Availability

All data generated or analyzed during this study are included in this published article.

## Abbreviations

GC: Gastric cancer
TG: Total gastrectomy
OTG: Open total gastrectomy
LDG: Laparoscopic distal gastrectomy
LTG: Laparoscopic total gastrectomy;
LTGD2: LTG combined with D2 lymphadenectomy
LDGD2: LDG combined with D2 lymphadenectomy
NOS: Newcastle–Ottawa scoring system
DFS: Disease-free survival
OS: Overall survival
OR: Odds ratio
MD: Mean difference
CI: Confidence interval
BMI: Body mass index
TNM: Tumor node metastasis
