# Supplementary material for: Outcomes of laparoscopic versus open total gastrectomy with D2 lymphadenectomy for gastric cancer: a systematic review and meta-analysis
Source: Eur J Med Res. 2022 Jul 18;27:124. doi: 10.1186/s40001-022-00748-2 (PMC9290297; doi:10.1186/s40001-022-00748-2)
Supplement: Supplementary file 1 — Additional file 1: Table S1. Details and characteristics of the studies included in this meta-analysis. [file 40001_2022_748_MOESM1_ESM.doc]

| **Additional file 1 Tables**  **Table S1.** **Details and characteristics of the studies included in this meta-analysis** | | | | | | | | | | | | | | | | | | |
| --- | --- | --- | --- | --- | --- | --- | --- | --- | --- | --- | --- | --- | --- | --- | --- | --- | --- | --- |
| **First author** | **Year** | **Country**  **/District** | **Journal** | **Study type** | **NOS score** | **Approach** | **Sample size** | **Age (years)** | **Gender**  **(M/F)** | **BMI**  **(kg/m^2^)** | **Tumor diameter(cm)** | **ASA class** | | | **Tumor stage** | | | |
|  |  |  |  |  |  |  |  |  |  |  |  | **1** | **2** | **3** | **Ⅰ** | **Ⅱ** | **Ⅲ** | **Ⅳ** |
| **Bo T** | 2013 | China | JGastrointest Surg | Case- control | 9 | LTGD2 | 117 | 54.5±10.6 | 82/35 | 21.1±3.0 |  |  |  |  | 6 | 40 | 52 | 19 |
|  |  |  |  |  |  | OTGD2 | 117 | 52.6±13.6 | 80/37 | 21.7±3.8 |  |  |  |  | 4 | 38 | 55 | 20 |
| **Chen, XZ** | 2017 | China | Oncotarget | Case- control | 7 | LTGD2 | 69 | 57.1±10.1 | 58/11 | 21.1±2.1 | 4.7±2.1 |  |  |  | 14 | 16 | 38 | 1 |
|  |  |  |  |  |  | OTGD2 | 268 | 60.7±9.9 | 209/59 | 22.6±3.0 | 6.0±2.5 |  |  |  | 24 | 59 | 169 | 16 |
| **Du J** | 2010 | China | Hepatogas- troenterology | Cross- sectional | 9 | LTGD2 | 82 | 60.4±18.5 | 54/28 | 22.3±2.6 | 5.4±1.4 |  |  |  | 3 | 36 | 42 | 1 |
|  |  |  |  |  |  | OTGD2 | 94 | 57.8±17.2 | 61/33 | 22.5±2.4 | 5.9±1.6 |  |  |  | 6 | 31 | 57 | 1 |
| **Eom BW** | 2012 | South Korea | Surg Endosc | Case- control | 8 | LTGD2 | 100 | 54.9±13.5 | 57/43 | 22.7±2.8 | 4.3±2.9 |  |  |  | 100 | 0 | 0 | 0 |
|  |  |  |  |  |  | OTGD2 | 348 | 58.7±11.5 | 254/94 | 23.8±2.9 | 4.4±3.0 |  |  |  | 348 | 0 | 0 | 0 |
| **Shu B** | 2016 | China | Journal of BUON | Case- control | 8 | LTGD2 | 136 | 65 (48-78) | 86/50 | 20 (16-26) |  | 112 | 16 | 8 | 21 | 67 | 48 | 0 |
|  |  |  |  |  |  | OTGD2 | 136 | 64 (42-73) | 92/44 | 21 (18-29) |  | 120 | 12 | 4 | 20 | 76 | 40 | 0 |
| **Guan G** | 2012 | China | Surg Endosc | Case- control | 6 | LTGD2 | 41 | 60.7±9.1 | 33/8 |  |  |  |  |  | 18 | 20 | 3 | 0 |
|  |  |  |  |  |  | OTGD2 | 56 | 57.8±9.9 | 40/16 |  |  |  |  |  | 25 | 25 | 6 | 0 |
| **Kawamura H** | 2019 | Japan | World J Srurg | cohort | 9 | LTGD2 | 46 | 64±10.4 | 36/10 | 22.8±3.0 |  | 15 | 27 | 4 |  |  |  |  |
|  |  |  |  |  |  | OTGD2 | 35 | 65.2±10.7 | 25/10 | 22.9±2.4 |  | 14 | 15 | 6 |  |  |  |  |
| **Kim HS** | 2013 | South Korea | Dig Surg | Case- control | 8 | LTGD2 | 139 | 56 | 134/73 | 24.1 | 3.2 |  |  |  | 0 | 0 | 139 | 0 |
|  |  |  |  |  |  | OTGD2 | 207 | 58 | 86/53 | 23.6 | 4 |  |  |  | 0 | 0 | 207 | 0 |
| **Kim KH** | 2011 | South Korea | J Korean Surg Soc | Cross- sectional | 8 | LTGD2 | 60 | 57.3±13.2 | 35/25 | 22.6±3.1 |  |  |  |  | 39 | 14 | 7 | 0 |
|  |  |  |  |  |  | OTGD2 | 60 | 56.7±12.4 | 36/24 | 22.8±3.3 |  |  |  |  | 40 | 13 | 7 | 0 |
| **Kim MG** | 2009 | Japan | Dig Surg | Cohort | 6 | LTGD2 | 63 | 55.9±12.2 | 43/20 | 22.7±2.5 | 3.8±2.1 | 45 | 15 | 3 |  |  |  |  |
|  |  |  |  |  |  | OTGD2 | 127 | 57.3±11.1 | 81/46 | 23.0±2.9 | 3.9±2.7 | 86 | 39 | 2 |  |  |  |  |
| **Lee MS** | 2013 | South Korea | Surg Endosc | Case- control | 9 | LTGD2 | 120 | 57.7±11.6 | 76/44 | 23.6±3.4 | 4.9±3.5 | 85 | 46 | 8 | 24 | 13 | 9 | 4 |
|  |  |  |  |  |  | OTGD2 | 228 | 58.2±12.1 | 156/72 | 22.6±3.1 | 7.4 ± 4.1 | 137 | 52 | 18 | 24 | 13 | 9 | 4 |
| **Lee SR** | 2014 | South Korea | Surg Laparosc Endosc Percutan Tech | Cross- sectional | 9 | LTGD2 | 34 | 61 | 25/25 |  | 3.3 |  |  |  | 22 | 7 | 5 | 0 |
|  |  |  |  |  |  | OTGD2 | 50 | 59 | 39/11 |  | 5 |  |  |  | 25 | 13 | 12 | 0 |
| **Lin JX** | 2014 | Taiwan | Chin Med J | Case- control | 8 | LTGD2 | 510 | 62.3±11.2 | 406/104 | 21.9±2.8 | 5.6±2.7 |  |  |  | 7 | 21 | 30 | 0 |
|  |  |  |  |  |  | OTGD2 | 586 | 60.4±10.2 | 463/123 | 22.0±2.1 | 5.9±2.9 |  |  |  | 9 | 21 | 28 | 0 |
| **Ramagem CA** | 2015 | Brazil | Arq Bras Cir Dig | Cross- sectional | 8 | LTGD2 | 47 | 57.8 | 34/13 | 23.8 |  |  |  |  |  |  |  |  |
|  |  |  |  |  |  | OTGD2 | 64 | 59.7 | 43/21 | 23.2 |  |  |  |  |  |  |  |  |
| **Topal B** | 2008 | Belgium | Surg Endosc | Cross- sectional | 7 | LTGD2 | 38 | 68 | 23/15 |  | 47 |  |  |  | 17 | 7 | 10 | 4 |
|  |  |  |  |  |  | OTGD2 | 22 | 69 | 17/5 |  | 30 |  |  |  | 7 | 7 | 6 | 2 |
| **Zhang GT** | 2017 | China | Surg Laparosc Endosc Percutan Tech | Case-control | 8 | LTGD2 | 69 | 69.4±10.5 | 38/31 | 20.9±2.1 |  | 41 | 27 | 1 |  |  |  |  |
|  |  |  |  |  |  | OTGD2 | 85 | 72.9±10.9 | 50/35 | 22.3±2.5 |  | 52 | 31 | 2 |  |  |  |  |
| **Feng XY** | 2021 | China | Frontiers in Oncology | Cohort | 8 | LTGD2 | 322 | 59 (50–66) | 218/104 | 22.1 (20.1–24.4) | 4 (3–5) |  |  |  | 20 | 123 | 179 | 0 |
|  |  |  |  |  |  | OTGD2 | 266 | 61 (53–67) | 199/67 | 22.1 (20.3–24.1) | 5 (3–7) |  |  |  | 19 | 48 | 199 | 0 |

Data are number, mean±standard deviation, or median(range).

LTGD2, laparoscopic total gastrectomy with D2 lymphadenectomy; OTGD2, open total gastrectomy with D2 lymphadenectomy; NOS, Newcastle-Ottawa scoring system; ASA, American Society of Anesthesiologists’; BMI, body mass index;
