# Supplementary material for: Outcomes of laparoscopic versus open total gastrectomy with D2 lymphadenectomy for gastric cancer: a systematic review and meta-analysis
Source: Eur J Med Res. 2022 Jul 18;27:124. doi: 10.1186/s40001-022-00748-2 (PMC9290297; doi:10.1186/s40001-022-00748-2)
Supplement: Supplementary file 2 — Additional file 2: Figure S1. Analysis comparing (A) blood loss, (B) times of analgesic medication injections, (C) time of first flatus per rectum, (D) time of initial diet, (E) postoperative hospital length of stay, and (F) subgroup analysis of operation time according to year of surgery. LTGD2, laparoscopic total gastrectomy with D2 lymphadenectomy; OTGD2, open total gastrectomy with D2 lymphadenectomy. Figure S2. Analysis comparing (A) postoperative mortality, (B) postoperative total complications, (C) postoperative incision-related complications, and (D) postoperative pulmonary complications. LTGD2, laparoscopic total gastrectomy with D2 lymphadenectomy; OTGD2, open total gastrectomy with D2 lymphadenectomy. [file 40001_2022_748_MOESM2_ESM.docx]

**Additional file Figures**


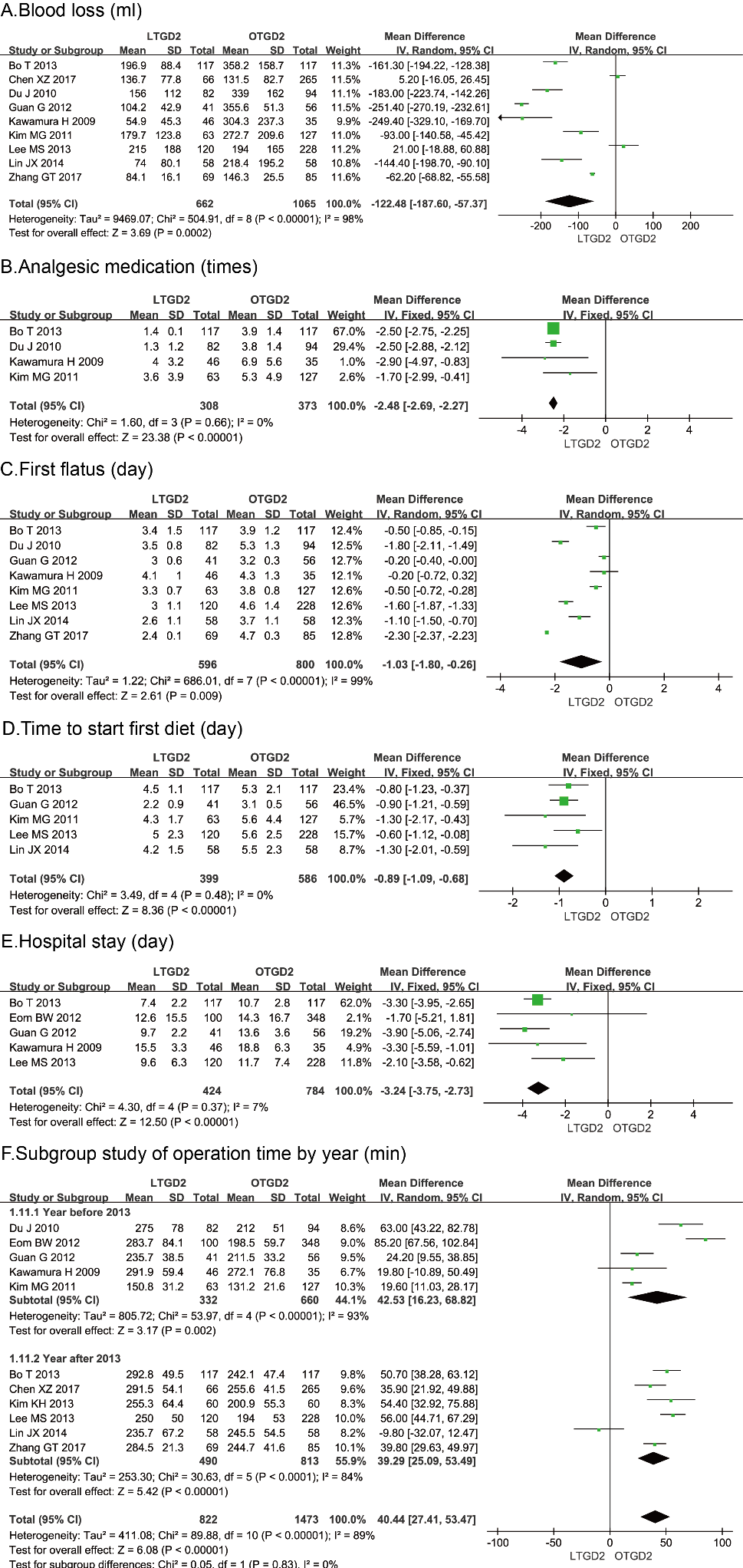


**Figure S1.** Analysis comparing (A) blood loss, (B) times of analgesic medication injections, (C) time of first flatus per rectum, (D) time of initial diet, (E) postoperative hospital length of stay, and (F) subgroup analysis of operation time according to year of surgery. LTGD2, laparoscopic total gastrectomy with D2 lymphadenectomy; OTGD2, open total gastrectomy with D2 lymphadenectomy.

**
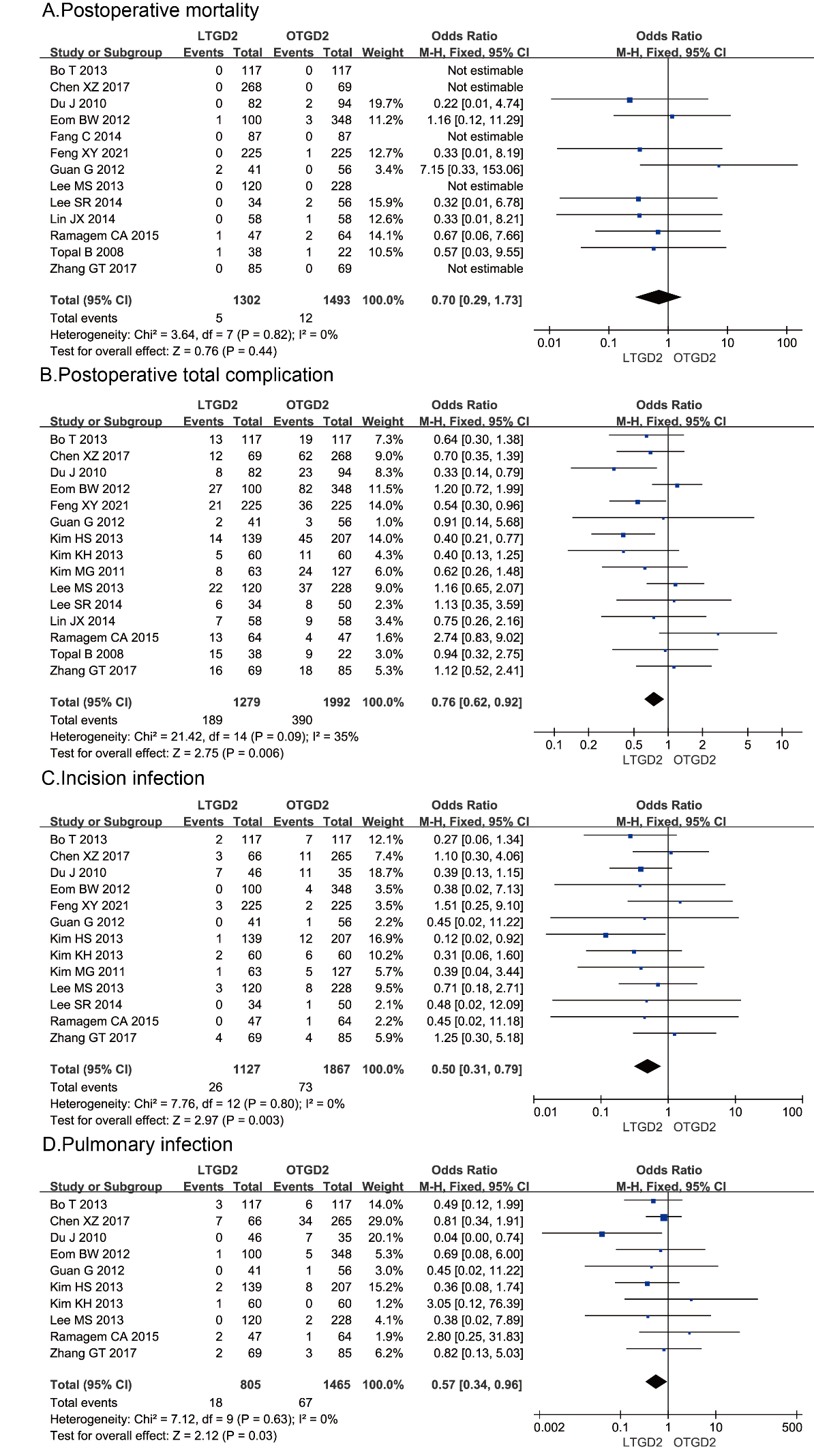
**

**Figure S2.** Analysis comparing (A) postoperative mortality, (B) postoperative total complications, (C) postoperative incision-related complications, and (D) postoperative pulmonary complications. LTGD2, laparoscopic total gastrectomy with D2 lymphadenectomy; OTGD2, open total gastrectomy with D2 lymphadenectomy.
